# Supplementary material for: Room-temperature second sound in isotopically pure graphite
Source: Nat Commun. 2026 Apr 3;17:4806. doi: 10.1038/s41467-026-70807-3 (PMC13219445; doi:10.1038/s41467-026-70807-3)
Supplement: Supplementary file 1 — Supplementary Information [file 41467_2026_70807_MOESM1_ESM.pdf]

# Supplementary Information for
# Room-temperature second sound in isotopically pure graphite

Zhikun Xie[1,2,3,†], Yifan Zhang[1,2,3,†], Xin Huang[4,†], Zhiwei Ding[5], Jie Wei[1,2,3], Difei Dong[1,2,3], Kun Cao[1,2,3], Tianshu Lai[1,2,3], Kenji Watanabe[6], Takashi Taniguchi[6], Xin Qian[7], Masahiro Nomura[4,*], Ke Chen[1,2,3,*]

[1]State Key Laboratory of Optoelectronic Materials and Technologies, School of Physics, Sun Yat-sen University, Guangzhou, 510275, China.
[2]Guangdong Provincial Key Laboratory of Magnetoelectric Physics and Devices, School of Physics, Sun Yat-sen University, Guangzhou, 510275, China.
[3]Center for Neutron Science and Technology, School of Physics, Sun Yat-sen University, Guangzhou, 510275, China.

[4]Institute of Industrial Science, The University of Tokyo, Tokyo, Japan.

[5]Department of Material Science and Engineering, Massachusetts Institute of Technology, Cambridge, MA, 02139, USA.

[6]Research Center for Materials Nanoarchitectonics, National Institute for Materials Science, Tsukuba, Japan.

[7]School of Energy and Power Engineering, Huazhong University of Science and Technology, Wuhan, 430074, China.

[†]These authors contributed equally to this work.

[*]Corresponding author. Email: chenk35@mail.sysu.edu.cn (Ke Chen) and nomura@iis.u-tokyo.ac.jp (Masahiro Nomura)

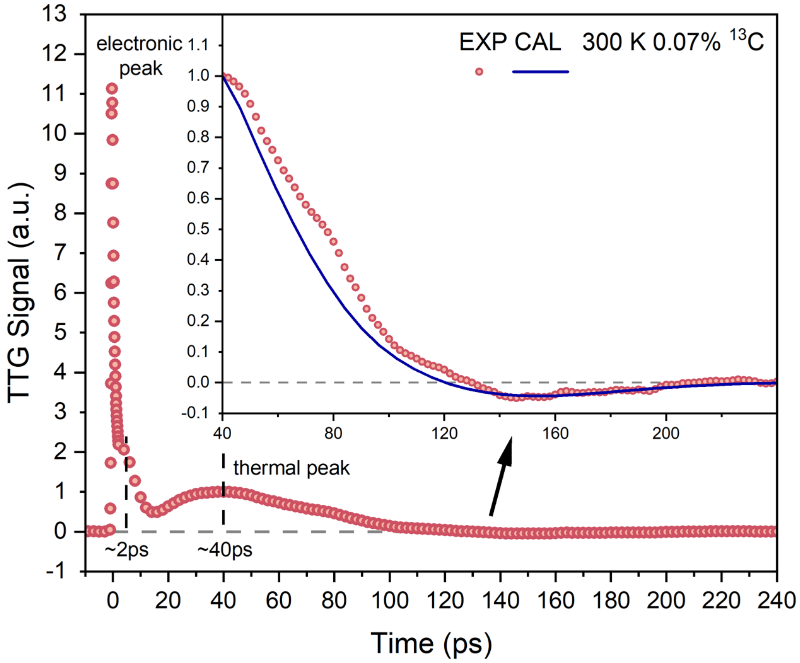


**Supplementary Fig. 1. The TTG Signal of whole dynamical process for isotope-purified graphite at 300 K with 0.95 $\mu$m grating period.** The TTG signal data illustrate the complete dynamical process following laser excitation. The data can be broadly divided into three stages of dynamical evolution. The electronic peak involves ultrafast carrier excitation, carrier relaxation and electron–phonon coupling (up to the first vertical dashed line, ~2 ps). The thermal peak corresponds to intrabranch thermalization of acoustic phonons (up to the second vertical dashed line, ~40 ps). After thermal peak, acoustic phonons relax with second sound.

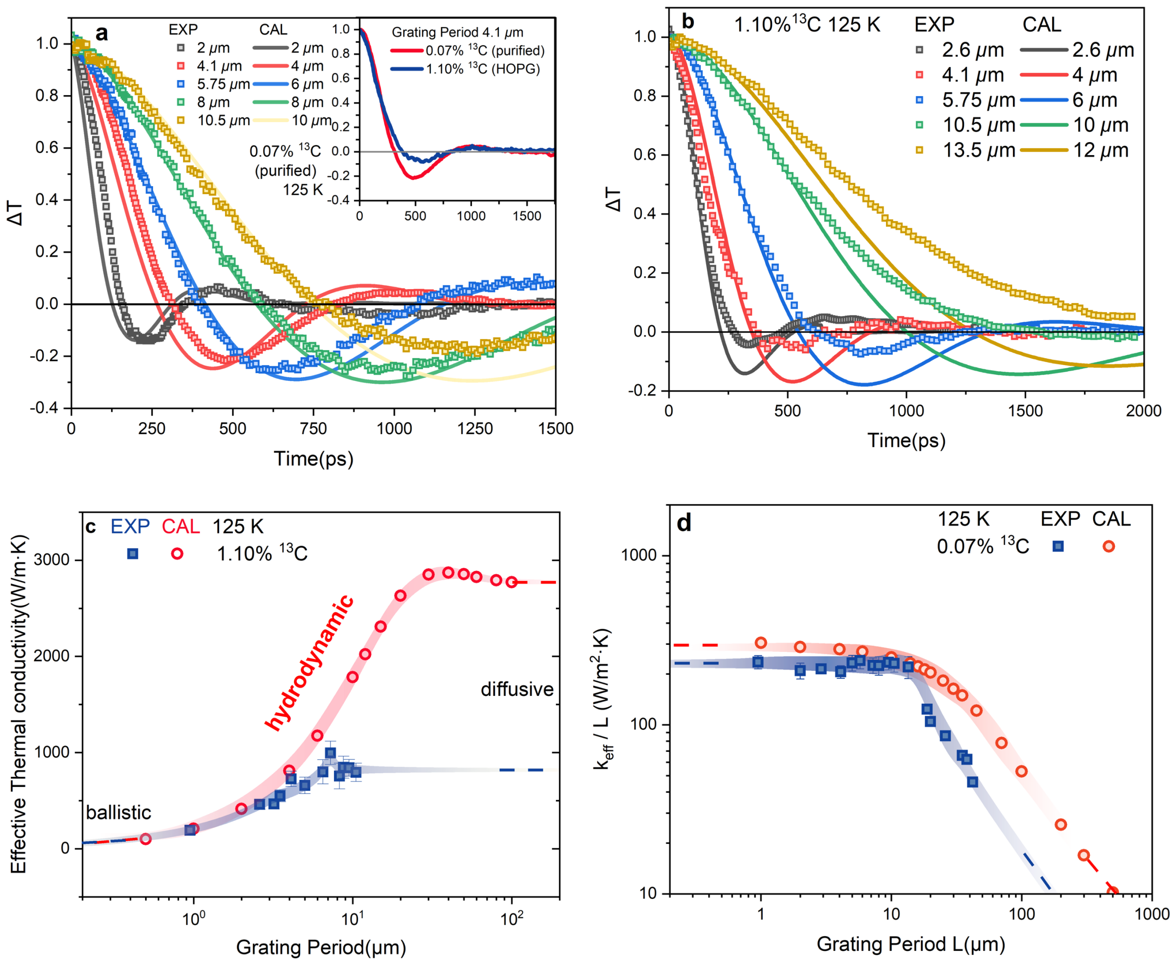


**Supplementary Fig. 2. TTG signals and analysis at different grating periods under 125 K.** **a**-**b**, Measured (squares) and simulated (lines) TTG signals of isotope-enriched graphite and HOPG with different grating period from 2-13.5 $\mu$m at 125 K. Inset on a: Isotope effect on second sound at grating period 4.1 $\mu$m. **c**, Thermal conductivity enhancement of HOPG by hydrodynamic phonons at 125 K. **d**, Normalized thermal conductivity of isotope-enriched graphite depending on the grating period.

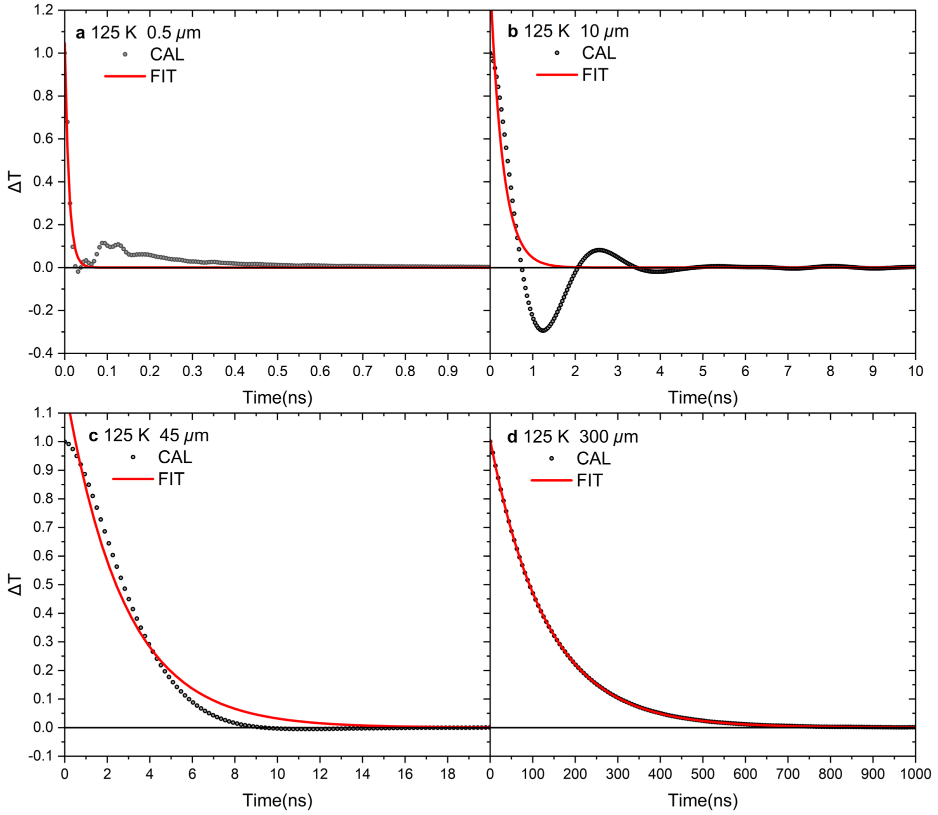


40

**Supplementary Fig. 3. The simulated temperature response (black dot) and fitted curve (red line) at 125 K with 1, 10, 45 and 300 $\mu$m grating period.** (**a**, **b** and **d**) correspond to conditions dominated by ballistic transport, hydrodynamic transport, and diffusive transport, respectively. **c**, shows the signal with the highest effective thermal conductivity.

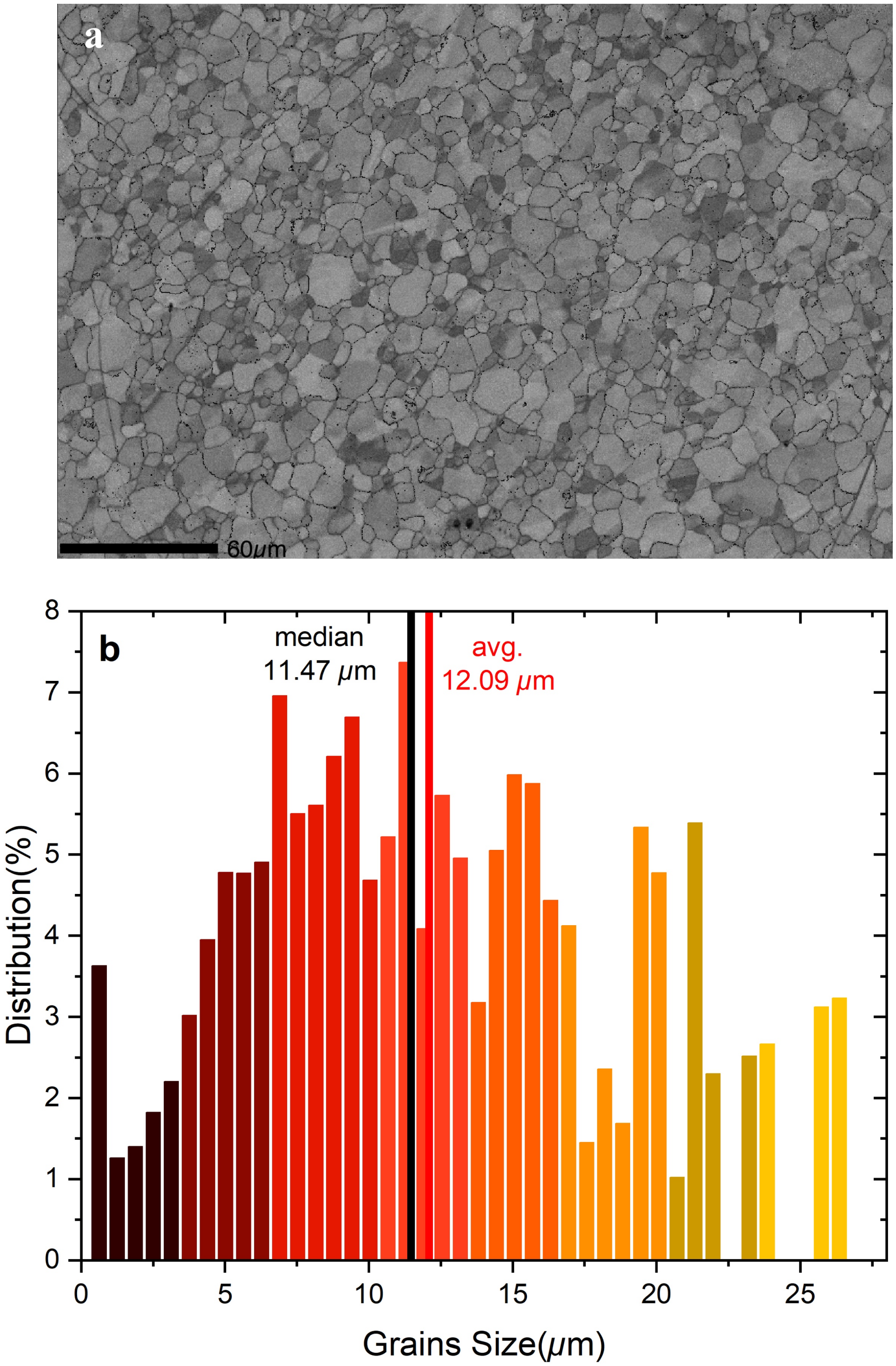


**Supplementary Fig. 4. Characterizations of the HOPG. a**, The EBSD pattern quality map of the HOPG surface showing the grain size in the basal plane. **b**, The statistical distribution of HOPG grain size shows that the average and median grain sizes are 12.09 $\mu$m and 11.47 $\mu$m, respectively.

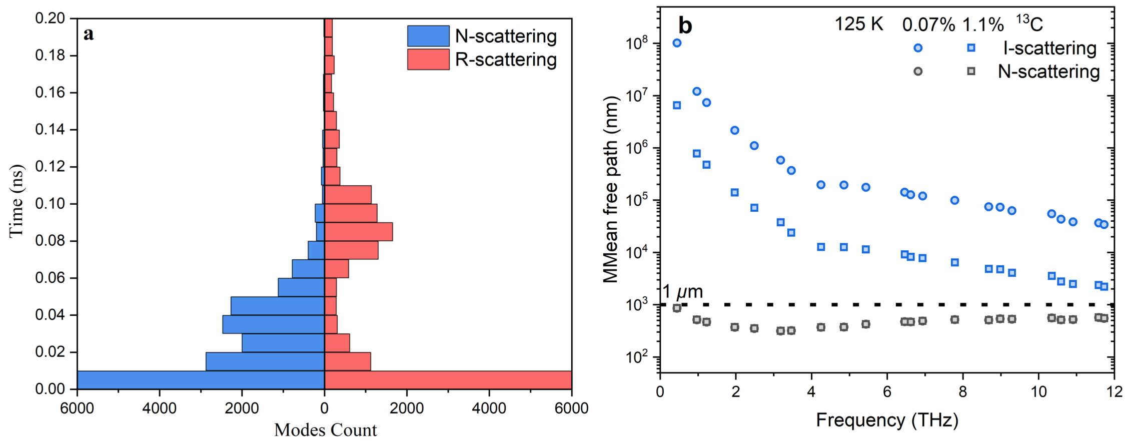


**Supplementary Fig. 5. Results of first-principles calculations by ShengBTE. a**, The distribution of resistive (R) scattering times and normal (N) scattering times for different phonon modes, with the relaxation times mainly ranging from 0 to 0.2 ns at 300 K. **b**, Comparison of mean free path for isotope-enriched graphite (squares) and HOPG (dots) at 125 K, isotopic (I) scattering and normal (N) scattering.

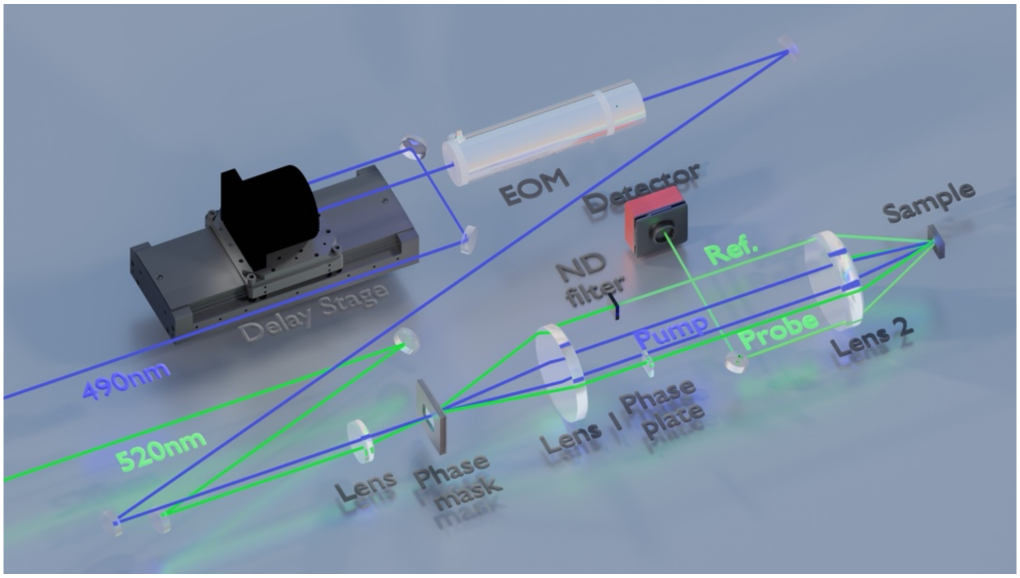


**Supplementary Fig. 6. Schematic of the TTG setup.**

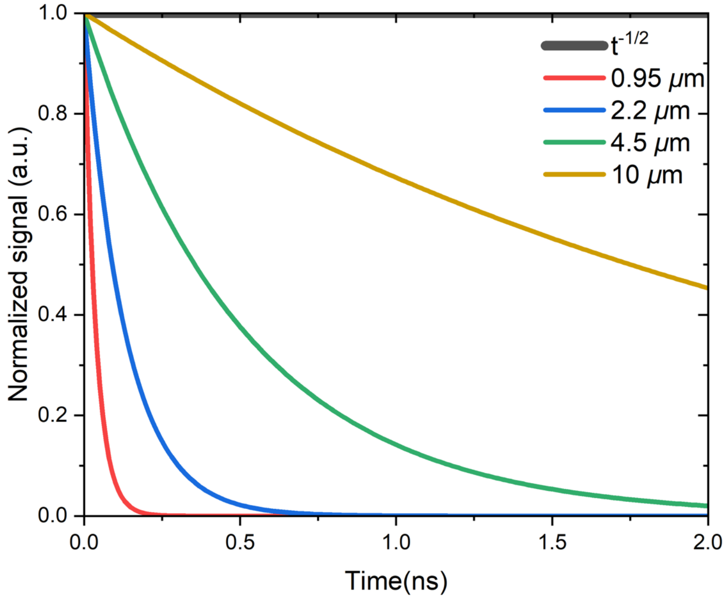


**Supplementary Fig. 7. Simulation of the normalized thermal expansion decay illustrating the different effects of cross-plane transport and in-plane transport on the TTG signal.** The heavy black curve shows the case where there is only cross-plane thermal transport, i.e., the in-plane thermal diffusion coefficient of graphite is intentionally set to be zero. The next four solid curves are simulated traces for grating periods of 10, 4.5, 2.2 and 0.95 $\mu$m with the consideration of in-plane transport by setting the in-plane diffusion coefficient to be graphite's value. Simulation results indicate that cross-plane thermal conductivity has no effect on the measured TTG signal.

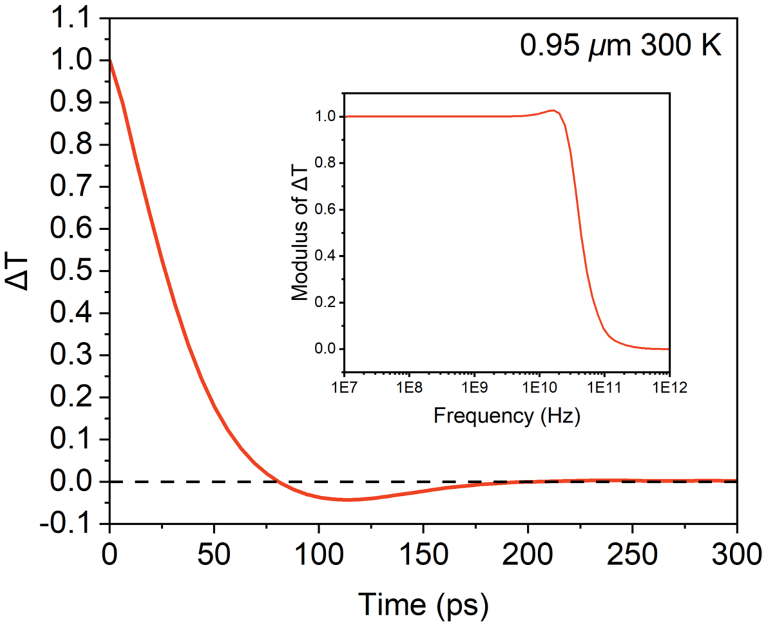


**Supplementary Fig. 8. Simulated TTG dynamics for isotope-purified (0.07% $C^{13}$) graphite**
70 **at 300 K with 0.95 $\mu$m grating period.**

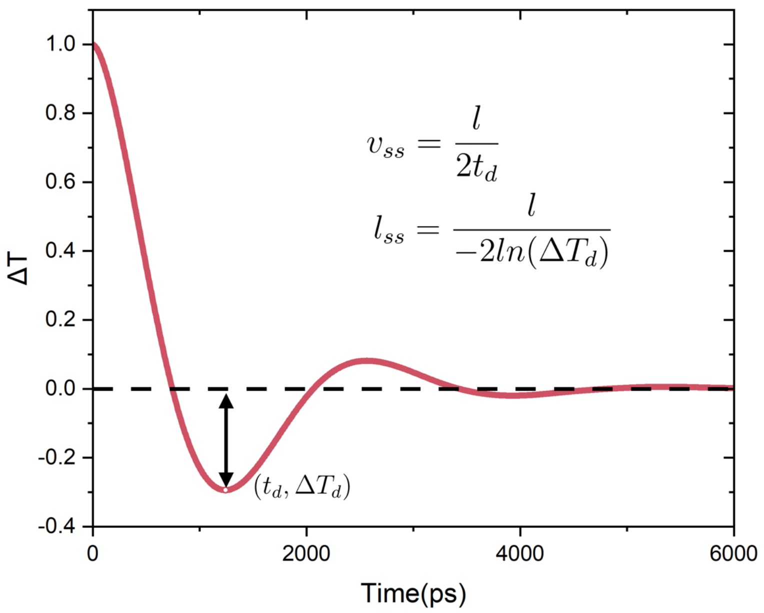


**Supplementary Fig. 9. The simulated temperature response at 125 K with a 10 $\mu$m grating period.** The first negative dip is the hallmark of wavelike thermal transport and its depth represents the second-sound strength.

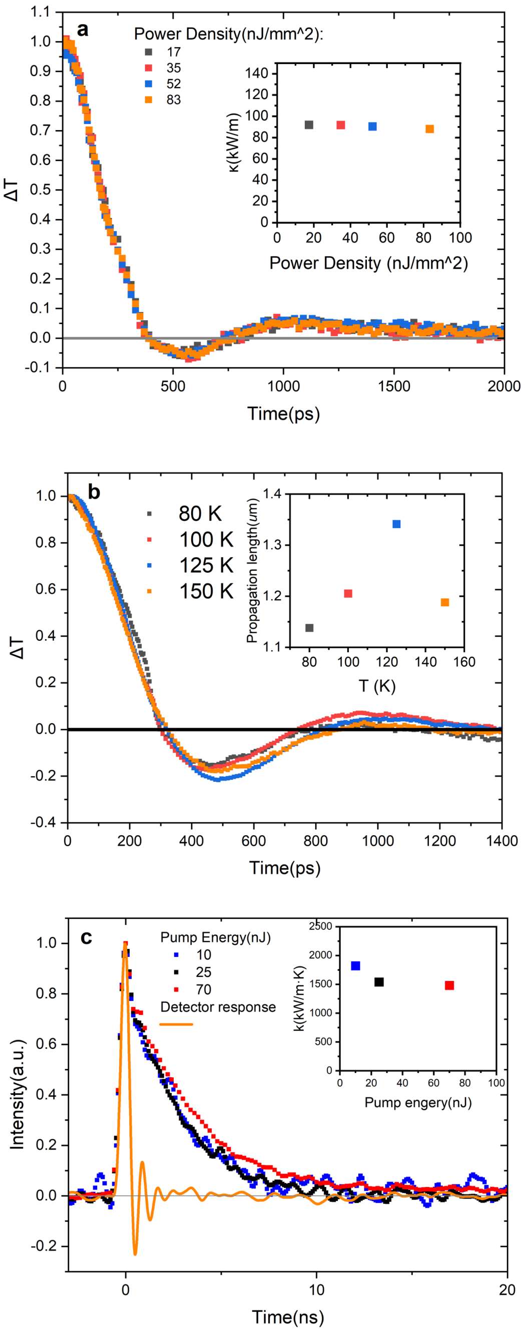


**Supplementary Fig. 10. The influence of power density, temperature, and pump energy on TTG experiments. a**, Measured TTG signals at 110 K on HOPG with a period L= 4.1 $\mu$m for pump power density from 20 to 90 nJ/mm$^2$ normalized to unity at the maximum. Insert: the effective conductivity with different power density. **b**, Measured TTG signals on isotope-enriched graphite with a period L= 4.1 $\mu$m for the temperature from 80 K to 150 K normalized to unity at the maximum. Insert: the propagation length of second sound extract from TTG signal, which shows the longest propagation length (means the strongest second sound effect) appears at 125 K. **c**, Dependence of the pump energy in HOPG at 300 K and a grating period of 10.4 $\mu$m, plotted together with the measured detector response. Insert: the effect of pump laser heating on the effective thermal conductivity.

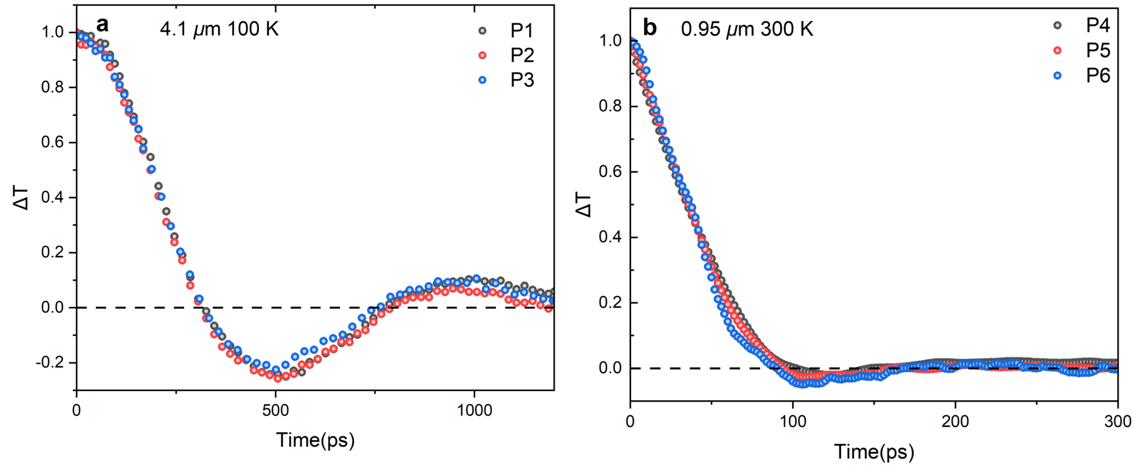


**Supplementary Fig. 11. Measured TTG signals of isotope-enriched graphite with different positions at grating period 4.1 $\mu$m 100 K (a), and 0.95 $\mu$m 300 K (b).**
