# Supplementary material for: Room-temperature second sound in isotopically pure graphite
Source: Nat Commun. 2026 Apr 3;17:4806. doi: 10.1038/s41467-026-70807-3 (PMC13219445; doi:10.1038/s41467-026-70807-3)
Supplement: Supplementary file 2 — Description of Additional Supplementary Files [file 41467_2026_70807_MOESM2_ESM.pdf]

Description of Additional Supplementary Files:

Supplementary Movie 1. Schematic diagram of the second sound excited by transient grating with the corresponding TTG signal.
